# Concurrent Validity of Equine Joint Range of Motion Measurement: A Novel Digital Goniometer versus Universal Goniometer

**DOI:** 10.3390/ani10122436

**Published:** 2020-12-19

**Authors:** Anna Bergh, Nicole Gandre Lauridsen, Amie Lamoreaux Hesbach

**Affiliations:** 1 Department of Clinical Sciences, University of Agricultural Sciences, 75007 Uppsala, Sweden; nega0001@stud.slu.se; 2 EmpowerPhysio, 2585CW The Hague, The Netherlands; amiehesbach@gmail.com

**Keywords:** goniometry, equine, digital, universal, joint range of motion, rehabilitation, physiotherapy

## Abstract

**Simple Summary:**

With the growth of the field of equine rehabilitation, there is an increased demand on rehabilitation clinicians to utilize simple and reliable objective outcome assessment tools. Goniometry, the measurement of joint angles, traditionally performed with a universal goniometer (UG), is a commonly utilized assessment tool in monitoring problems of the musculoskeletal system, as well as the progression of rehabilitation interventions. Goniometry has been demonstrated to be of value, being both reliable and valid, in humans and other animal species. There are different types of goniometers, each of which has some benefit, but varies in accessibility, cost, and user-friendliness. This study examined the use of a novel digital goniometer (DG) in the measurement of angles of two joints in a horse, and comparing the measurements to those taken with a UG. The study demonstrated significant differences in range of motion for the carpus and fetlock joint, a 2–3° difference comparing measurements on a sedated horse with corresponding radiographs, a “fair” to “excellent” inter-tester reliability and a “fair” to “almost perfect” intra-tester reliability. The novel DG was found to be easier to use than the UG. In conclusion, the novel DG may serve as a simple tool for measuring joint motion in equine rehabilitation patients.

**Abstract:**

Goniometry is the measurement of joint angles with a conventional universal goniometer (UG) or a digital goniometer (DG). The UG is validated for use in dogs and cats. However, it demands both of the user’s hands when measuring. To avoid this, a novel type of DG has been developed, simplifying measurement by offering single-hand usage. The objective of this study is to examine the concurrent validity of the DG. The study consists of measurement with the DG and UG for flexion of the carpus and fetlock on ten horses, and with the DG in flexion and neutral positioning of the fetlock on a sedated horse and corresponding radiographs, intra- and inter-tester reliability and a survey on user-friendliness. The data were analyzed with ANOVA and intraclass correlation coefficient (ICC), with a significance of *p* < 0.05. The study showed significant differences in range of motion for the carpus and fetlock joint, a 2–3° difference comparing measurements on a sedated horse with corresponding radiographs, a “fair” to “excellent” inter-tester reliability and a “fair” to “almost perfect” intra-tester reliability, and the DG was easier to use than the UG. In conclusion, the novel DG may serve as a simple tool for measuring joint motion in equine rehabilitation patients.

## 1. Introduction

With the rapidly increasing interest in equine rehabilitation, there is a subsequent demand for simple and reliable tools to assess the efficacy of rehabilitation modalities and training. As interventions in rehabilitation often focus on problems from the musculoskeletal system, one important factor is the assessment of joint motion. This is especially true as an adequate range of motion is a prerequisite for optimal functional mobility for independent daily activity. A restriction in joint motion can contribute to changes in joint loading and result in additional impaired function. Therefore, the method can be particularly useful for measurements in osteoarthritis. Thus, objective measurement of joint range of motion (ROM) is essential for the evaluation of joint health, as well as in the development and progression of treatment and rehabilitation plans.

Goniometry is the measurement of joint angles performed with a goniometer. Goniometry may be a useful outcome measure in monitoring the severity of orthopedic disease in horses and the progression of rehabilitation interventions, as it has been shown in humans and other animal species [1,2,3,4,5,6,7,8,9,10]. It can be performed with different types of goniometers—universal goniometers (UG), parallelogram goniometers, electrogoniometers, computerized goniometers, smartphone goniometry applications, and digital goniometers (DG). Each type of goniometer has some benefit, however, issues such as accessibility and cost suggest that the UG remains the equipment of choice for joint angle measurement in clinical practice [11,12,13]. UG has been validated to different degrees in several species, including dogs [2,14,15], cats [16], and humans [17,18]. However, the validation of its use in horses is limited [19,20]. Although UG is cheap and measurements are fairly quick, it requires the use of both of the tester’s hands. This amount of dexterity and coordination can be a challenge in a large animal such as the horse. Furthermore, the measurement of range of motion in joints such as the equine carpus may require a UG with longer arms than that of commercial UGs [20].

In human rehabilitation clinical practice, the use of DGs has started to replace UG. DG claims to have several advantages over UG, one of which is that it can be administered single-handedly. A novel DG is the EasyAngle, based on an inertial measurement unit technology, thus replacing the inclinometer, a tool considered to be the most accurate in the measurement of knee joint angles when compared to using smartphone applications and short- and long-arm Ugs [21]. However, DGs have not been validated for use in horses. Therefore, the objective of the present study is to examine the concurrent validity of a novel DG, the EasyAngle, and to examine the goniometer´s user-friendliness. This is performed by (1) determining whether there were differences in goniometric data between a novel DG and a UG for the measurement of maximal passive flexion of the fetlock and carpus, (2) determining whether there were differences in goniometric data between a sedated horse and corresponding radiographic images of the same horse, (3) assessing intra- and inter-tester reliability, and (4) a compilation of survey results investigating the goniometers´ user-friendliness. The hypothesis was that there would be no significant difference in measurement results between the novel DG and the UG, no significant difference between measurement results for the novel DG on a horse and corresponding radiographic images of the same horse, that there is good inter- and intra-reliability, and that the novel DG was considered to be more user-friendly than the UG.

## 2. Materials and Methods

### 2.1. Animals

Ethical approval for animal experiments was received from the ethics committee in (information withdrawn as a result of blinding) and performed with informed consent of the owners. The study was conducted on ten university-owned Standardbred trotters (eight mares and two geldings, age 15 ± 4 years, weight 564 ± 68 kg). The horses were deemed sound by the university veterinarian and were kept under the same conditions, with daily outdoor activities in a paddock.

### 2.2. Goniometers

**Figure 1:**
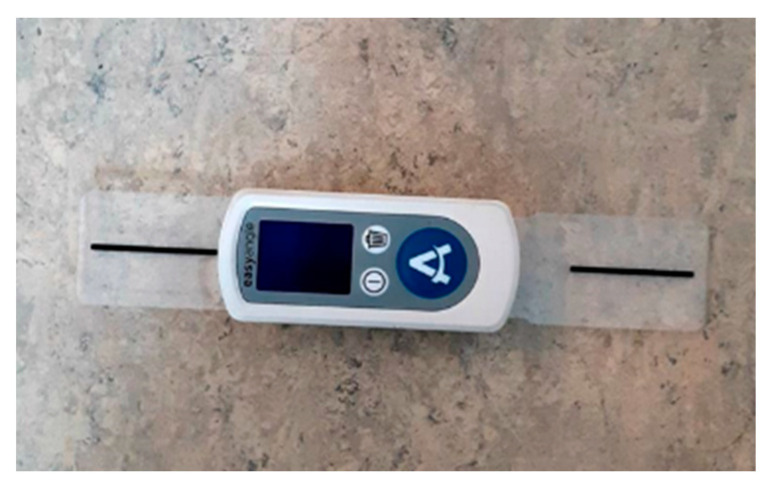
Digital goniometer, EasyAngle. (Photo: Nicole Gandre Lauridsen).

Two types of goniometers were utilized in the study. The UG (Whitehall Manufacturing, City of Industry, CA, USA) has a transparent plastic 360° face, two movable arms, and a 1° gradation. The novel DG, the EasyAngle (Meloq AB, Stockholm, Sweden), is a battery-powered device based on inertial measurement unit technology, combining a three-axis accelerometer and a three-axis gyroscope for continuous determination of the goniometer position in space (Figure 1). The DG was originally designed for use on humans, and calculates the angle formed by the start position and end position of the device, relative to the proximal and distal component of the joint angle measured. The accuracy is 1 degree within the 180-degree range.

### 2.3. Testers

The goniometric measurements were performed by three final-year students enrolled in the Bachelor of Veterinary Nursing program and by one veterinarian. The students had limited experience in the clinical use of goniometry.

### 2.4. Experimental Procedure

The experiments were conducted at (information withdrawn as a result of blinding) during two afternoons in February 2019. Prior to the commencement of the study, the examiners were instructed in the use of both goniometers through written instruction, instructional videos, and training sessions for the study protocol. During the group training sessions, there was a demonstration followed by practical training for each examiner. The testing session did not begin until each examiner was confident with the protocol.

The measurements with both types of goniometers were performed according to anatomical landmarks described by Jaegger et al. [14], Liljebrink and Bergh [19], and Adair et al. [20]. The examiners had access to the reference articles during the two testing sessions. The measurements with the DG were performed according to the manufacturer’s instructions (Meloq AB, Stockholm, Sweden). The DG was placed parallel to the body part (ray or “arm”) being measured and the recording button was depressed; this position was registered by the device as the start position. The goniometer was then placed on the second body part (ray or “arm”), the recording button was depressed again, and the digital goniometer calculated the angle at the vertex formed by the start position and end position of the device.

**Figure 2:**
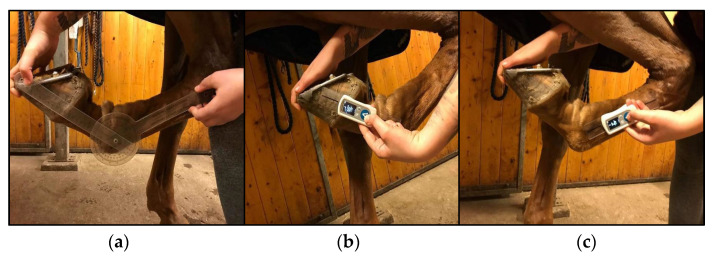
(**a**) Measurement of the fetlock joint with a universal goniometer. Measurement of the fetlock joint with a digital goniometer for the (**b**) start and (**c**) end positions (photo: Nicole Gandre Lauridsen).

The horses were taken from their stalls and walked for five minutes before the start of the experiment. The horses were standing in the stable outside their stall during the measurements. The horses were only manually restrained, so as to mimic the clinical condition during measurements. The examiners were instructed to measure the maximal passive flexion of the horse’s right carpus and fetlock (Figure 2). Maximal flexion was defined as a position where the angle between the bones of the joint was maximally decreased. The examiners were instructed to use one type of goniometer, measure one joint at a time, repeat each measurement three times, and then change joints. The order of the joint and type of goniometer was randomized. The examiner was instructed to put the joint in a neutral position between each measurement, and to ensure that the adjacent joints were in neutral positions. The reading of the result was performed with the joint in its end position, by an independent recorder who documented the angle in whole degrees. Then, the examiner changed the type of goniometer and repeated the procedure. The maximal passive flexion range of motion was measured in triplicate with both the UG and the DG in a randomized order, resulting in three measurement values for each examiner and joint. There was approximately 30 s between each repeated measurement and a 5 min duration for each joint, and, thus, 5 min with each type of goniometer.

### 2.5. Radiography

**Figure 3:**
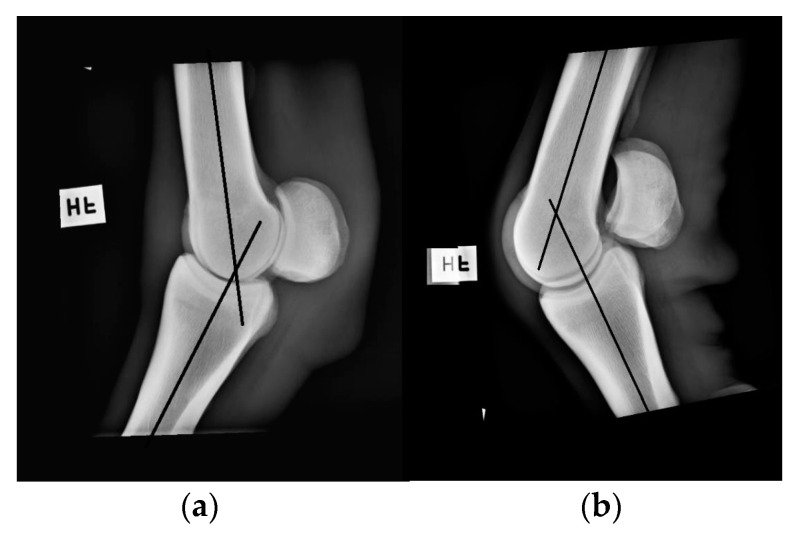
Radiographic images of the fetlock joint in a (**a**) neutral position and in (**b**) flexion (photo: Nicole Gandre Lauridsen).

Neutral position and flexion of the right forelimb fetlock joint was measured with the DG on one healthy Standardbred trotter, separate from previous measurements. The goniometric measurements were conducted with the horse being sedated (0.3 Ml detomidin 10 mg/Ml + 0.3 Ml butorphanol 10 mg/Ml i.v.), and were done immediately before standard lateromedial radiographs (CPI Indico IQ, Ontario, Canada) were taken with the horse standing squarely (Figure 3). The measurements were then conducted on the radiographic images, serving as a “gold standard” [20].

### 2.6. Survey of User-Friendliness

A questionnaire with statements regarding the user-friendliness of the two goniometers was designed and thereafter answered by each examiner within the test group, following completion of the second testing session. The questionnaire consisted of five identical statements for each goniometer, with answers on a Likert scale ranging 1–5 (1 = strongly disagree, 5 = strongly agree). The statements were as follows: In my future professional life, it is likely that I will use the EasyAngle/UG often; I think that the EasyAngle/UG is easy to use; I think that it was easy to learn how to use the EasyAngle/UG; I consider the EasyAngle/UG to be accurate and reliable; and I feel confident in using the EasyAngle/UG.

### 2.7. Statistical Analyses

Power analyses with a power of 0.80 and *p* < 0.05 were performed based on data from Liljebrink and Bergh [19] and Adair et al. [20]. The sample size was determined to be eight subjects. Descriptive statistics (mean + SD) were calculated for all goniometric data—a total of 480 measurements resulting in 160 mean values. Nonparametric tests (Wilcoxon Sign-Rank and Mann–Whitney) and an ANOVA test were used to determine whether there was a significant difference in joint ROM between horses, investigators, and/or goniometric techniques. To evaluate the intra- and inter-tester reliability for the four testers´, the intra-class correlation coefficients (ICCs) were calculated and classified according to Landis and Koch [22]. Statistical software (Microsoft Corporation, Redmond, Washington, USA; Statsoft Scandinavia AB, Uppsala, Sweden; R) were used for analyses and the significance level was set at *p* < 0.05.

## 3. Results

The goniometric data from all of the horses and testers are shown in Table 1 and Table 2.

**Figure 4:**
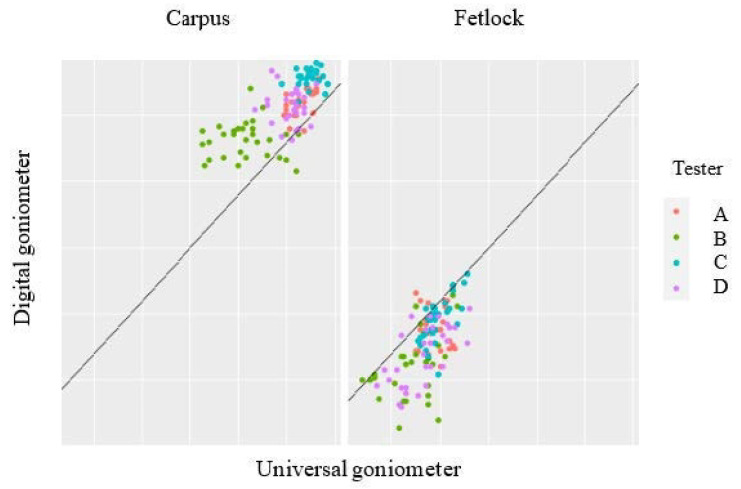
Concurrent validity of the digital goniometer, using the universal goniometer as the reference standard.

When comparing the values for all horses and testers, there was a significantly higher value for the measurement of fetlock flexion with the UG (116 ± 12°) compared with the DG (106 ± 8°), with *p* < 0.001. When comparing the values for all horses and testers, there was a significantly lower value for the measurement of carpal flexion with UG (26 ± 10°) compared with DG (40 ± 12°), with *p* < 0.001. The concurrent validity of the novel DG, using UG as the reference standard, is shown in Figure 4. There was a good correlation between the assessors, however, with a systematic overestimation for the measurement of the carpal joint and an underestimation for measurement of the fetlock joint.

The results from the measurements on a sedated horse and its corresponding radiographic images showed that there was 2–3° difference depending on the joint position. Measurements of the sedated horse´s fetlock in a neutral position was 218° and on the corresponding radiographs was 216°. Measurements of the sedated horse´s fetlock in a flexed position was 138° and on the corresponding radiographs was 135°.

The inter-tester reliability was fair for measuring the fetlock joint (ICC DG = 0.39; UG = 0.25) and excellent for measuring the carpus (ICC DG = 0.77, UG = 0.77). The intra-tester reliability was substantial to almost perfect for measuring the fetlock joint (ICC DG = 0.76–0.90; UG = 0.66–0.90) and moderate to excellent for the carpus (ICC DG = 0.0.47–0.66; UG = 0.33–0.81), according to the classification set by Landis and Koch [22].

The results from the survey on the goniometers´ user-friendliness is presented in Table 3. There was one significant difference when comparing answers, the testers rated the DG as easier to use (*p* = 0.006).

## 4. Discussion

To the best of our knowledge, this is the first study to investigate the validity of the novel EasyAngle DG for the assessment of joint range of motion in horses. The results of the goniometric data show similar values for flexion of the fetlock joint and the carpus joint, as previously reported by Liljebrink and Bergh [19], as well as by Adair et al. [20]. However, the present study shows divergent results compared with those reported. In the present study, the inter-tester reliability for flexion of the fetlock joint was fair (ICC 0.25) and excellent (ICC 0.77) for flexion of the carpus, compared with almost perfect in the Adair study (ICC 0.946; 0.97). As for the intra-tester reliability, the present study shows ranges between ICC 0.33–0.90 and the Adair study shows ranges between ICC 0.95–0.99. That study, however, assessed sedated horses, and a suggestion is that the variance in measurement values is higher for measures of non-sedated horses. A difference in measurement values has been reported in the study by Liljebrink and Bergh [19], with higher goniometric values and ICCs when measuring anaesthetized horses compared with awake horses. However, the influence of sedation on goniometry values is debated, as studies on dogs have shown that there is both a difference and that there is no difference between sedated and non-sedated values [13,23]. Nevertheless, the experimental set-up resembles that of a clinical situation, and the results of the study indicate that the measurements of the UG and DG are reliable regardless.

When considering the validation process of the EasyAngle DG in the present study, the criteria for the most suitable reference standards were discussed. The selection of a reference standard is less restricted, as goniometry in clinical practice is used to quantify and monitor changes over time and not to register an absolute value. Because of the reported reliability and widespread use, a number of studies of different species have used UG as the reference standard for validating other types of goniometers [24,25]. Therefore, in the present study, the results from a new DG were compared to those from a UG. The study shows significant differences between the measurement values for DG and UG regarding maximal passive flexion of the fetlock and the carpus. These results indicate that DG and UG are not interchangeable; instead, it suggests that the same type of goniometer should be used in subsequent measures of the same horse. This is especially important when assessing the progression of a treatment or training session.

A difference in results based on the type of goniometer used has also been shown for canine subjects. Freund et al. report, in a study on measurement with two smartphone-based applications and a DG (HALO, HALO Medical devices, Australia) compared to a UG conducted on four cadavers, that the mean coefficient of variation was the lowest for the UG (4.88%), followed by the two smartphone-based applications, and, finally, by the DG (12.71%) [25].The same study reports that UG had the highest (0.97) and DG the lowest (0.78) correlation with radiographic measurements. This is in accordance with the results from Adair et al., showing a correlation of 0.78 for carpal flexion and 0.70 for fetlock joint flexion with radiographic measurements [20]. As the previous study had compared the use of an UG on sedated horses with corresponding radiographic images, the focus in the present study was to investigate the intra- and inter-tester reliability and user-friendliness of DG. However, to enable some comparison, measurements were performed on one sedated horse and its corresponding radiographic images, showing a 2–3° difference.

As a result of the lack of studies validating the EasyAngle DG on animals, inferences must be made from human studies. The intra- and inter-rater reliability of the EasyAngle DG were proven to be excellent to almost perfect (ICC 0.63–1.0) in a study by Fröjd, investigating the measurement of both active and passive hip range of motion [26]. Another study looking at the measurement of knee joint range of motion also showed excellent inter- and intra-rater reliability [27]. In the present study, there was a tendency that goniometric data from the novice testers had a higher variability.

A potential limitation of the present study is that the majority of the test group had limited experience in goniometry. However, studies have shown that there was no significant difference in measurement values between a novice and an experienced tester [27,28]. In the future, it would be useful to compare the results obtained from a group of non-experienced testers with experienced ones. There was no significant difference in experienced user-friendliness between the two goniometers, except that significantly more respondents stated that it was easier to use DG. One possible reason is that DG can be used with one hand, which offers a significant advantage when working with animals, especially those that are large or difficult to handle or restrain.

## 5. Conclusions

There were significant differences in the registration values for the flexion of the fetlock joint and the carpus joint when comparing measurements with the novel DG and UG. This implies that the goniometers are not interchangeable; instead, that the measurements should be conducted on one animal with one type of goniometer. The inter-tester reliability was fair to excellent and the intra-tester was fair to almost perfect, and more respondents ranked DG easier to use compared with UG. Thus, the novel DG may serve as an easy tool for measuring joint range of motion in equine rehabilitation patients, preferably when using the goniometer within the same horse and with the same tester.

## Figures and Tables

**Table 1:** Joint angles of fetlock joint flexion, as performed on ten horses by four testers and with two types of goniometers. Values are presented as mean ± standard deviation, and in degrees.

Horse	Tester A		Tester B		Tester C		Tester D	
	DG-Fetlock	UG-Fetlock	DG-Fetlock	UG-Fetlock	DG-Fetlock	UG-Fetlock	DG-Fetlock	UG-Fetlock
1	121 ± 3	102 ± 3	129 ± 1	128 ± 3	134 ± 2	110 ± 5	123 ± 5	104 ± 3
2	111 ± 8	100 ± 3	114 ± 6	110 ± 6	128 ± 4	112 ± 7	115 ± 2	108 ± 0
3	116 ± 6	108 ± 2	136 ± 3	105 ± 0	131 ± 5	121 ± 6	113 ± 3	103 ± 1
4	108 ± 2	107 ± 1	135 ± 5	122 ± 6	136 ± 6	118 ± 3	111 ± 3	106 ± 1
5	98 ± 3	97 ± 2	101 ± 2	100 ± 9	115 ± 2	95 ± 5	123 ± 5	104 ± 3
6	106 ± 5	104 ± 2	120 ± 3	102 ± 4	120 ± 9	101 ± 5	115 ± 2	108 ± 0
7	117 ± 2	95 ± 1	125 ± 5	112 ± 7	113 ± 3	105 ± 10	98 ± 7	94 ± 5
8	112 ± 0	104 ± 0	144 ± 5	111 ± 9	109 ± 6	108 ± 4	103 ± 2	102 ± 0
9	99 ± 2	108 ± 3	124 ± 2	116 ± 2	114 ± 6	105 ± 2	107 ± 5	104 ± 2
10	104 ± 3	98 ± 1	119 ± 2	108 ± 5	106 ± 4	94 ± 6	106 ± 1	102 ± 2
Mean ± SD	109 ± 8	102 ± 5	125 ± 12	111 ± 9	120 ± 11	107 ± 9	111 ± 8	104 ± 4

Notes: DG—digital goniometer; UG—universal goniometer.

**Table 2:** Joint angles of carpal joint flexion, as performed on ten horses by four testers and with two types of goniometers. Values are presented as mean ± standard deviation, and in degrees.

Horse	Tester A		Tester B		Tester C		Tester D	
	DG-Carpus	UG-Carpus	DG-Carpus	UG-Carpus	DG-Carpus	UG-Carpus	DG-Carpus	UG-Carpus
1	27 ± 3	37 ± 1	37 ± 2	60 ± 3	12 ± 4	37 ± 3	16 ± 3	36 ± 6
2	19 ± 3	28 ± 0	27 ± 7	54 ± 3	27 ± 5	45 ± 1	12 ± 3	27 ± 2
3	25 ± 5	36 ± 0	45 ± 7	37 ± 3	29 ± 8	43 ± 4	10 ± 4	28 ± 2
4	32 ± 3	40 ± 1	41 ± 4	52 ± 3	29 ± 3	49 ± 2	16 ± 5	36 ± 0
5	30 ± 5	35 ± 1	37 ± 4	68 ± 7	25 ± 4	35 ± 4	14 ± 2	30 ± 2
6	24 ± 4	29 ± 0	37 ± 2	61 ± 14	31 ± 8	35 ± 4	14 ± 3	31 ± 1
7	22 ± 2	35 ± 5	42 ± 5	65 ± 8	25 ± 6	32 ± 1	23 ± 2	30 ± 6
8	27 ± 3	35 ± 2	48 ± 2	71 ± 4	31 ± 7	38 ± 2	15 ± 2	32 ± 2
9	23 ± 3	32 ± 3	35 ± 3	49 ± 8	16 ± 10	34 ± 2	15 ± 2	31 ± 4
10	27 ± 1	40 ± 0	35 ± 3	54 ± 10	33 ± 3	35 ± 0	16 ± 2	28 ± 4
Mean ± SD	26 ± 4	35 ± 4	38 ± 6	57 ± 10	26 ± 7	38 ± 6	15 ± 3	31 ± 3

Notes: DG—digital goniometer; UG—universal goniometer.

**Table 3:** Mean and standard deviation for questionnaires regarding the user-friendliness for the digital goniometer and universal goniometer (N = 4).

Statement (1 = Strongly Disagree; 5 = Strongly Agree)	DG	UG	*p*-Value
In my future professional life, it is likely that I will use the EasyAngle/UG often.	3.3 ± 1.0	3.0 ± 0.0	0.22
I think the EasyAngle/UG is easy to use.	4.3 ± 0.5	2.5 ± 0.6	0.006
I think that it was easy to learn how to use the EasyAngle/UG.	3.8 ± 1.0	3.3 ± 0.5	0.18
I consider the EasyAngle/UG to be accurate and reliable.	4.0 ± 0.8	3.5 ± 0.6	0.18
I feel confident in using the EasyAngle/UG.	3.0 ± 0.0	3.3 ± 0.5	0.39

Notes: DG—digital goniometer; UG—universal goniometer.
